# Impact of the COVID-19 pandemic on breast cancer patient pathways and outcomes in the United Kingdom and the Republic of Ireland – a scoping review

**DOI:** 10.1038/s41416-024-02703-w

**Published:** 2024-05-04

**Authors:** Lynne Lohfeld, Meenakshi Sharma, Damien Bennett, Anna Gavin, Sinéad T. Hawkins, Gareth Irwin, Helen Mitchell, Siobhan O’Neill, Charlene M. McShane

**Affiliations:** 1 Queen’s University Belfast, Centre for Public Health, School of Medicine, Dentistry & Biomedical Sciences, Royal Victoria Hospital, 247 Grosvenor Road, Belfast, BT12 6BA Northern Ireland UK; 2 Northern Ireland Cancer Registry, Centre for Public Health, School of Medicine, Dentistry & Biomedical Sciences, Queen’s University Belfast, Mulhouse Building, Grosvenor Road, Belfast, BT12 6DP Northern Ireland UK; 3 Belfast Health and Social Care Trust, 51 Lisburn Road, Belfast, BT9 7AB Northern Ireland UK

**Keywords:** Breast cancer, Health services

## Abstract

The COVID-19 pandemic brought unplanned service disruption for breast cancer diagnostic, treatment and support services. This scoping review describes these changes and their impact in the UK and the Republic of Ireland based on studies published between January 2020 and August 2023. Thirty-four of 569 papers were included. Data were extracted and results thematically organized. Findings include fewer new cases; stage shift (fewer early- and more late-stage disease); and changes to healthcare organization, breast screening and treatment. Examples are accepting fewer referrals, applying stricter referral criteria and relying more on virtual consultations and multi-disciplinary meetings. Screening service programs paused during the pandemic before enacting risk-based phased restarts with longer appointment times to accommodate reduced staffing numbers and enhanced infection-control regimes. Treatments shifted from predominantly conventional to hypofractionated radiotherapy, fewer surgical procedures and increased use of bridging endocrine therapy. The long-term impact of such changes are unknown so definitive guidelines for future emergencies are not yet available. Cancer registries, with their large sample sizes and population coverage, are well placed to monitor changes to stage and survival despite difficulties obtaining definitive staging during diagnosis because surgery and pathological assessments are delayed. Multisite longitudinal studies can also provide guidance for future disaster preparedness.

## Introduction

Approximately 60,000 people are diagnosed with breast cancer annually in the United Kingdom (UK) and the Republic of Ireland (RoI) [1, 2]. Services for screening, diagnosing, treating and follow up of patients provided through national health care services varied by country. During both the initial phase of the COVID-19 pandemic in 2020 and throughout subsequent peaks in transmission, various restrictions were implemented that limited and/or changed how breast cancer was diagnosed, treated and managed in much of the world [3], including the UK and RoI. Given the importance of early detection and treatment of cancer, there is concern over how COVID- related service delays may affect cancer patients now and in the future regarding stage at diagnosis, prognosis and mortality [4]. Because potentially life-changing decisions about cancer patients’ care have been made rapidly without the benefit of prior experience, there has been a sudden increase in studies examining possible pandemic impacts on breast cancer services and patients. To better understand the full impact of the COVID-19 pandemic on breast cancer diagnosis, treatment and patient outcomes in the UK and RoI, we conducted a scoping review that would examine findings from several studies conducted in these countries.

## Methods

Scoping reviews aim to rapidly map key concepts in a research area that have not been studied comprehensively and identify research gaps in the existing literature [5].

The present scoping review used Arksey and O’Malley’s [6] framework, minus the last step of expert validation of findings due to resource constraints. Generally, this type of review does not include a critical appraisal of the constituent material. The Preferred Reporting Items for Systematic reviews and Meta-Analyses extension for Scoping Reviews (PRISMA-ScR) checklist was used to report the review findings [7].

**Fig. 1:**
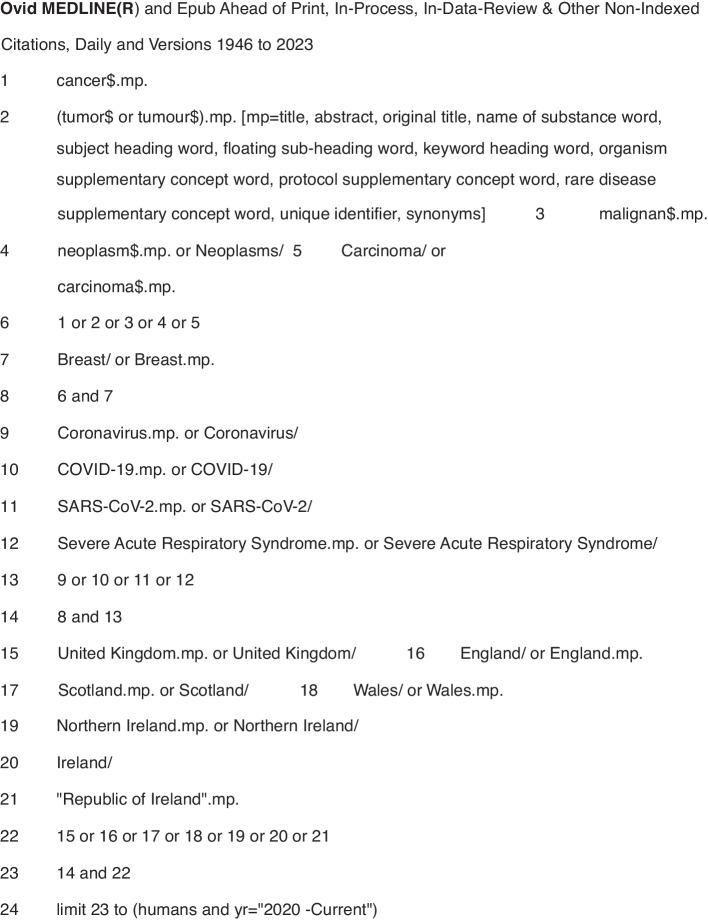
Search strategy used in Ovid Medline. Symbols: $ is a wildcard to expand the search term and find both British and American spellings of the same word. .mp. means multi-purpose for an Advanced search without specifying a particular field. / means the term preceding it is from the MeSH headings in MEDLINE.

A systematic search was conducted on five electronic databases -- PubMed, Medline, Web of Science, Embase and PyschInfo -- using key words and MeSH headings for breast cancer services and outcomes in the countries of interest (Fig. 1). Inclusion criteria were publication in English in a peer-reviewed journal between 1 January 2020 and 31 August 2023, and reporting on primary data collected in the UK or RoI. Papers excluded from this report either did not meet the inclusion criteria or: described an intervention other than healthcare system changes or patient outcomes directly related to breast cancer; provided data from multiple locations without separately identifying results from the UK and/or the RoI; or were systematic reviews, conference abstracts, or proceedings, or unpublished (grey) literature. A hand search of the reference lists of each included paper was done.

Results from each electronic database were imported into the Covidence systematic review software [8], an online tool to support doing systematic reviews that automatically removes duplicate entries. Title and abstract screening was done independently by three reviewers (CM, LL, MS) who discussed differences of opinion about papers’ eligibility until reaching consensus. After removing ineligible studies, the remaining papers were downloaded and independently screened by the reviewers against the inclusion and exclusion criteria. Any differences of opinion were resolved through discussion. The reviewers included a cancer epidemiologist, a public health professional and a medical anthropologist.

Data were extracted from the selected papers and entered into an Excel spreadsheet containing information on the bibliography (authors, title, journal, publication date), study aims and design, geographic location, and key findings (Table 1, Supplementary Material). Results were then organised thematically to describe the impact of the COVID-19 pandemic on the organisation of breast cancer services, referrals/diagnosis and number of cases, and treatment.

A study protocol was not written and registered. The scoping review is part of a larger study on the impact of COVID-19 on breast cancer services in Northern Ireland.

## Results

**Fig. 2:**
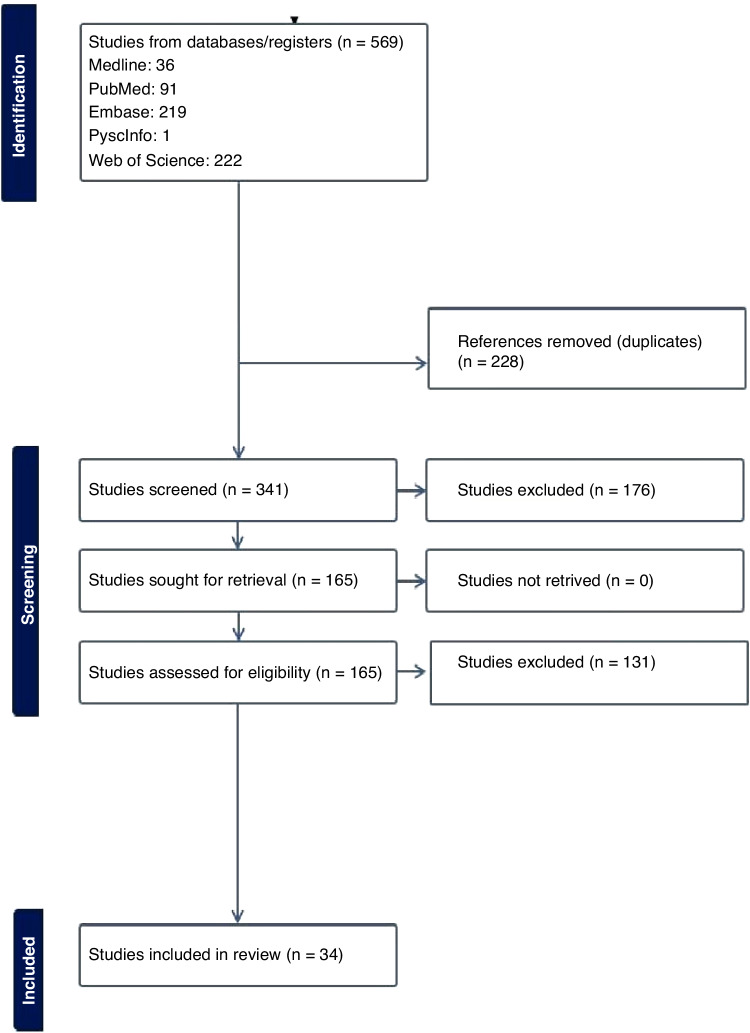
Prisma flowchart.

The electronic database search returned 569 studies. Following duplicate removal (*n* = 228), over half (176/341, 51.6%) of the screened studies were deemed irrelevant, leaving 165 studies for full-text review. Of these studies, 129 were excluded, primarily because they were published as a conference abstract. The remaining 34 papers used in the review included 16 studies conducted in England [9–24], four in Scotland [25–28], three in [29–31] Wales, one in Northern Ireland [32], three in the UK [33–35], one in Ireland [36] and six that used data from multiple countries which included at least one site in the UK and/or [37–42] RoI. No additional studies of interest were identified in the hand search of reference lists (Fig. 2).

### Impact of the COVID-19 pandemic on the organisation of breast cancer services

During the first wave of the COVID-19 pandemic (March–April 2020), population-based breast cancer screening programs were paused in many jurisdictions, including the UK and RoI. There were also major changes in how members of multidisciplinary teams (MDTs) met to develop treatment plans for breast cancer patients [11, 37]. One study in an English hospital tested the acceptability of video-conferencing MDT meetings with participants attending in person or from a remote location. After overcoming minor technical difficulties (e.g. uninterrupted access to online meetings, ensuring participants had the necessary equipment to attend meetings remotely) all the participants indicated that online meetings were acceptable or their preferred mode of communication [11]. Another study surveyed breast pathologists in the UK and RoI who reported their MDTs often met in small virtual meetings [37]. Although nearly three-quarters of them indicated their workload and productivity decreased during the pandemic, 36% reported improved efficiency [37]. No study reported on the optimal balance between virtual and in-person meetings.

Three studies examined changes made to referral pathways to breast clinics or units in response to the COVID-19 pandemic [14, 19, 23]. One study, using data from England’s National Health Service, reported a 28% decline in referrals for suspected breast cancer during the first six months of 2020 compared to the same period in 2019 [14]. Another research group reported an even greater decline (−35%) in the number of women attending a one-stop rapid breast clinic in England during the initial lockdown (March-April 2020) compared to June-July that year [23].

A study reported on rapid adaptations made by a London-based breast cancer service in line with The Royal College of Surgeons guidelines to reduce the risk of COVID-19 [19]. Examples include providing space to maintain the recommended two metre distance between people; fewer appointments plus longer time between them to allow for thorough cleaning of surfaces; following stricter criteria for urgent referrals; and conducting routine follow-up appointments over the phone. In addition, although diagnostic imaging with ultrasound and mammogram continued to be available, all routine surveillance imaging was deferred for three months. Operations were conducted by small teams of specialists who travelled to a “cold” (free of COVID-19 cases) private hospital [19]. Virtual appointments quickly became the norm for many patients. However, as noted by one research team [14] this increased the potential for greater inequality of access to care by the elderly or people of lower socioeconomic status.

Several studies observed smaller-than-expected numbers of attendees at breast cancer screening and treatment centres [9, 23, 26, 41]. This was noteworthy given the association between early detection through screening and the potential to reduce treatment needed potential to reduce treatment needed with better patient outcomes. Reasons for the downtrend in attendance ranged from centres issuing fewer invitations to ensure adequate time between appointments for cleaning equipment [26], to women declining invitations to be screened due to fears of being exposed to SARS-CoV-2 when in a healthcare facility [9].

Other investigators focused on how to effectively restart breast screening programs [18, 26]. A Scottish study described the benefits of using a phased approach for this, giving priority to high-risk women, followed by recalling program participants, issuing new invitations to women of screening (age 50–70 years or older) or those who had missed or cancelled earlier appointments [26]. In another study [18], researchers in London investigated whether switching from sending women invitations to attend a specific appointment (“timed appointments”) to having them book their sessions (“open appointments”) would reduce the backlog of unscreened eligible women. Both invitation types were used between September 2020 and March 2021, allowing researchers to conduct a natural experiment to examine which approach had the greatest response [18]. The authors found significantly fewer women responded to the open than to the timed invitation (−7.5%) and estimated that if timed invitations were exclusively used approximately 12,000 more women would have attended screening and about 100 more women with breast cancer would have been detected [18].

### The Impact of COVID-19 on referrals, diagnoses and numbers of patients with breast cancer

A major concern regarding COVID-19 is the possible effect that delaying or modifying diagnosis and treatment would have on patients, including those with symptomatic disease, and the potential for excess breast cancer deaths. An English study used national data to estimate the impact of curtailing screening during the first lockdown on predicted breast cancer deaths from 2020 to 2029. The authors estimated up to 687 additional deaths in that 10-year period [13]. Routinely collected NHS England data were used to compare referral patterns and time to first treatment for breast cancer during the pandemic (first half of 2020) compared to the same period in 2019 [14]. Results showed a 28% decrease in diagnostic services and 16% of patients receiving their first treatment. They also noted that hormonal therapy, administered in tablet form, had become a frequent alternative to surgery – the mainstay treatment for breast cancer before the pandemic [14].

Five studies reported on the number of new breast cancer cases during the pandemic in Wales and England [10, 20, 22, 29, 30], with results varying widely by location and time period. For example, a Welsh study [29] found a 2% reduction of cases in April 2020 compared to the same period in 2019, whereas an English study reported a 17.9% reduction in March-April 2020 versus 2019 [20]. Three other English studies [10, 22, 30] reported reductions in the number of new diagnoses ranging from 19.1% to 29.5%.

Four studies [10, 22, 28, 30] reported on changes in disease severity or stage of cancer at diagnosis, finding clear evidence of stage migration to more advanced cases attributed to delayed diagnosis of new cases.

Most breast cancer diagnoses are confirmed through pathology. A study [32] from Northern Ireland compared the number of pathologically-diagnosed (PD) breast cancer cases before the pandemic (2017–2019) with numbers during the early pandemic. The researchers found 105 fewer breast cancer cases in 2020, with the greatest reductions in the early months (−40% in April, −52% in May) [32]. A UK-based study [39] compared population-based cancer registry data from Northern Ireland, Scotland and Wales, with sharp declines in the number of patients with breast cancers in each country (−53.5% in Northern Ireland, −45.3% in Scotland, −43.5% in Wales). The finding of fewer PD-confirmed cases of breast cancer was also reported in a study [36] conducted in the histopathology departments of two university hospitals in Northwest RoI. The larger hospital reported a decline of 21.5% and 14.4% in the first six months of 2020 compared to 2019 for samples from small biopsy diagnostic procedures and cancer resection cases, respectively [36].

The Impact of COVID-19 on Treatment: As noted in several studies [17, 21, 24, 25, 31, 34, 35, 40, 42], efforts to reduce the risk of exposure to COVID-19 SARS-CoV-2 for patients and healthcare providers resulted in fewer surgical, radiotherapy or systemic treatments of breast cancer patients. There were also changes to facility procedures used to reduce the amount of time patients were potentially exposed in medical facilities.

Four studies [17, 21, 40, 42] addressed changes to surgical treatment during the pandemic. One of them reported on an international web-based poll with over 100 oncological surgeons that included practitioners from the UK. In both Scotland and England, surgical priority was given to patients with ER-negative disease first followed by those with HER2-positive disease, and that neoadjuvant chemotherapy was to be given following standard criteria. In England, there was also a recommendation to focus on providing minimal treatment via day surgery, with neoadjuvant chemotherapy to be reserved for patients whose disease was deemed to be inoperable [42].

Another study found a 34% decline in “radical surgery with curative intent” for breast cancer done in a large London cancer centre from March to September 2020 compared to 2019 [40]. Surgical practices were also altered, such as having procedures done by only consultant surgeons because junior doctors were redeployed to COVID-19-related duties during the first two months of the pandemic [40]. Another study [21], conducted at the Oxford University Hospitals in England, reported the unit followed recommendations from the Association of Breast Surgery and did not perform immediate or delayed breast reconstruction between the start of lockdown (23 March 2020) and the end of May despite the known psychological and physical benefits of immediate reconstruction for many women. In two English hospitals surgical procedures continued during the pandemic but at greatly reduced numbers compared to 2019, with declines in both immediate and delayed reconstructive surgeries. Patients also had significantly shorter hospital stays post-surgery [17].

Widespread changes to radiotherapy regimens also occurred during the pandemic. Earlier, conventional treatment entailed giving 40–42.5 Gray (Gy) units of radiation divided into 15 treatments or ‘fractions’ (F) over a 3-week period. During the pandemic, this protocol was replaced in many centres with a hypofractionated radiation regimen consisting of a smaller amount of radiation divided into five treatments given over a week (26GyF5). The impetus for this was the publication of guidelines by The Royal College of Radiologists [43] recommending this shift based on findings from the FAST-Forward non-inferiority trial [44] and the B-MaP-C study [45].

Radiation oncology teams quickly complied, reporting increases during the pandemic (up from 13 to 48% in Wales, [31] and 0.2% to 60.6% in England [24] and 2.7% to 46.1% in Scotland [27]), as well as during the pandemic. (up from <1% in February to 70% in April 2020 in a study from England and Wales [38]).

Another four studies [12, 25, 34, 35] examined changes in systemic anticancer treatment (SACT), noting this was used as a “bridging” or pre-operative treatment while waiting for breast cancer surgery during the pandemic. One study from England [42] found a 33% decrease in the number of patients registered for SACT immediately after the initial lockdown (April–June 2020) compared to numbers from September 2019 to February 2020.

Modifying or halting cancer treatments was also identified in the B-Map-C study [45] -- a multicentre national project involving 64 breast units in the UK – which reported that 59% of all breast cancer patients received a “COVID-altered” management plan (e.g. interrupted neoadjuvant chemotherapy or bridging endocrine therapy instead of surgery) during the initial pandemic period from March 16 to May 8, 2020 [34]. In contrast, a study conducted in a hospital in England found that 56% of women being treated for breast cancer chose to continue SACT despite clear recommendations from the National Institute for Health and Care Excellence (NICE guidelines) [46] that such treatment should stop during the pandemic to reduce the risk of exposure to SARS-CoV-2 in a hospital setting. Some authors suggest this indicates that many patients feared the effects of not treating their cancer more than they feared COVID-19 [35].

## Discussion

The studies included in this scoping review identified unprecedented changes to breast cancer services over a short period of time. During the COVID-19 pandemic people with non-urgent stage disease typically diagnosed via screening (e.g. breast, colorectal or cervical cancer) saw a decrease in the number of new cases due to temporary closures or reduced healthcare facility capacity [47]. This pattern is borne out by population-based data from national cancer registries reporting 11–21% fewer cases diagnosed during the pandemic in ROI [1] and the UK [47–51] despite a year-on-year increase in cases.

Evidence exists for both overdiagnosis and benefits from diagnosing breast cancer through screening. [52] It is inevitable that pauses in population-based screening programs during the pandemic resulted in fewer early-stage cancers being diagnosed. However, the long-term deleterious effects of halting screening programs during health emergencies has yet to be determined. None of the included papers in the review were able to provide evidence of direct harm to patients due to reduced detection rates, despite evidence of more advanced disease on detection. In fact, one study clearly indicated that such delays may have less of an impact than commonly believed for surgeries conducted <12 weeks after diagnosis [53]. The full extent of harm caused to people with breast cancer can only be answered once enough data comparing outcomes related to delayed services before, during and after the pandemic have been analysed.

The studies examined in this scoping review point to efforts made to continue to offer timely services, including early detection and treatment, with a focus on identifying high-priority patients based on tumour- and patient-related characteristics [52] taking into account availability of healthcare personnel and services during the pandemic [54, 55]. Recovery plans for future emergencies [56] must help implementers decide whether to prioritise rapid resumption of breast screening programs or preserve symptomatic diagnostic services [4] while taking measures to minimise the risk of communicable disease transmission for patients and staff in breast clinics [33].

There are also lessons to be learned about the benefits of rapidly incorporating evidence from high-quality studies, such as the FAST-FORWARD clinical trial demonstrating the effectiveness of hypofractionated radiotherapy for eligible patients, into clinical practice during the pandemic [44]. Another modification was to preferentially offer neoadjuvant therapy over surgery for triple negative or HER2+ patients during the pandemic. This likely was to reduce through flow in chemotherapy departments, thereby reducing the risk of exposing immunocompromised patients to SARS-CoV-2 [28], although future studies will be needed to determine the effectiveness and long-term impact of this change.

It is also important to adapt international guidelines to fit local conditions [57]. Factors to consider would be how to continue providing services while safeguarding patients and staff given local resources, what criteria to use when identifying high-priority patients during times of reduced service availability, ensuring that resources are available for increased use of remote/virtual consultations and MDT meetings, as well as developing locally acceptable approaches to phasing in full services post-emergency [58].

Other recommendations for breast cancer programs focus on ways to avoid undertreatment with neoadjuvant therapy and, in some cases, providing breast-conserving operations [54] in “clean” surgical sites even during a health emergency. Benefits from continuing to operate include ensuring that surgical trainees continue developing their skills, and so there will be more clinicians available to help reduce the backlog of patients once operations resume [54]. Second, it should reduce the number of women experiencing unnecessary anxiety and depression, which have been found in patients waiting considerable time for their breast surgery [59, 60]. Third, as recommended by the British Association of Plastic, Reconstructive and Aesthetic Surgeons in the UK [61], resuming breast reconstruction quickly can help prevent unnecessarily long or repeat procedures due to tissue change that occurs over time after a mastectomy, which increase hospital stay and potentially the risk of exposure to SARS-CoV-2. However, the link between length of stay and infection rates has yet to be proven. It is also important to consider the surgical environment, as noted by The Royal College of Surgeons in May 2020 [62]. This included guidelines for the “four Ps”: the Place for surgeries should be reconfigured to provide a safe setting for patients and clinicians; People should return to their pre-COVID work in order to reduce the backlog of elective cases; PPE should be made available for all staff; and no major surgery for Positive Tests (i.e. if patients test positive for COVID-19) except for life-, (limb- or sight-saving procedures) [21]. Future research will determine if these actions are effective in reducing the risk of infection with SARS-CoV-2.

Public awareness campaigns should also be delivered that includes the clear communication [55] for people with relevant symptoms to seek medical care promptly [57], even at the height of a pandemic or other emergency.

Looking to the future, it will be important to fund research on the long-term impact of delayed or interrupted breast cancer services on patient outcomes such as cancer incidence, stage, tumour size and ultimately survival [15, 16, 63]. For instance, previous studies have found survival differences for women with breast cancer only if the delay in services was longer than 12 weeks [53, 62]. Several of the papers in this review reported results from single-site retrospective studies [62], which is problematic because it is not possible to generalise their findings to other settings or populations. This problem can be alleviated by using data from multicentre investigations and national cancer registries. However, there are issues with obtaining timely information from registries. First, many registries do not have data on cancer recurrences, which makes it difficult to accurately assess the impact of health emergencies. Efforts to address this gap are being led by the European Network of Cancer Registries [58]. Second, cancer registries use patient-level data retrospectively after they are received and cleaned. Further delays in producing reports were identified during the COVID-19 pandemic, when monitoring was curtailed due to registry staff working off-site or allocated to pandemic-related duties. This delayed data analysis and report preparation. Several registries have reported they can address such problems in the future by adopting novel methods for more quickly assessing the impact of modified and interrupted services during health emergencies [64].

Although studies have documented changes in the breast cancer service profile and outcomes during the COVID-19 pandemic, there is no evidence available on whether these measures helped minimise the spread of the SARS-CoV-2 infection. Further research is also needed on the long-term effects of changes to breast cancer services for patients who had advanced disease on initial presentation or whose treatment was delayed [65]. Findings from such studies can be used to update models that predict the number of excess deaths from breast cancer due to interrupting care [66].

Studies are needed to provide insights into the following: how health emergencies affect the cost and availability of services while considering how closely they follow disaster preparedness guidelines; more accurate estimates of cancer risks and consequences for designing optimal recovery strategies [59–61]; and recommendations on how to address the backlog of breast cancer cases requiring surgery or other treatment in a timely and safe manner [67, 68].

Perhaps the most important gap in current literature on the impact of COVID-19 on breast cancer services and patients is research to document the patient voice and experience, as well as research to evaluate improvements in service timeliness and efficiency during the pandemic which has not compromised patient satisfaction and safety.

Health emergencies like the COVID-19 pandemic are the norm rather than the exception. There are valuable lessons to be learned from existing studies conducted in the short time since the end of the pandemic. There is also a need to pool data and design future studies to provide more evidence to guide future plans on how to best meet the needs of women (and men) with breast cancer during future emergencies. It is impossible to completely prepare for future health emergencies, especially those involving novel pathogens. Evidence extrapolated from other infectious diseases, and recommendations by experts (e.g. oncologists, pathologists and patients) on how to better manage cancer treatments in future emergencies should be considered [69].

### Strengths and limitations

To our knowledge, this is the first scoping review to examine the published literature on the impact of the COVID-19 pandemic on breast cancer services and patient outcomes in the UK and RoI. The review was conducted following a strict protocol carried out by three reviewers with conflicts resolved by consensus.

Because of the short time since the end of the pandemic, findings from more definitive, longitudinal, population-based studies were not available to include in this review. The authors also chosen not to review the grey literature because there is no established guidelines for producing a rigorous review of material that does not meet the level of evidence expected by healthcare providers, commissioners and policymakers.

Another limitation is the wide variation in study design and context, such as the stage of the pandemic when data were being collected, among the studies included in the review. Of particular concern was the large number of retrospective, single-centre studies with data from a relatively homogeneous population, making it difficult to generalise findings beyond a particular study setting.

This scoping review presents a coherent picture of current published knowledge on the impact of the COVID-19 pandemic on breast cancer services and patient outcomes in the UK and RoI. It also recommends ways to fill current knowledge gaps on this topic, summarising findings from studies documenting changes made to breast cancer services provided during the COVID-19 pandemic in the UK and RoI.

The long-term impact of these changes are still unknown. Lessons for future disaster preparedness will come from large-scale, multisite studies and cancer registries using data collected before, during and after the pandemic. Results will be useful for developing guidelines to help reduce the impact of future medical emergencies on people with breast cancer and on healthcare systems and providers.

### Supplementary information

Table 1: Characteristics of 34 included studies

Preferred Reporting Items for Systematic reviews and Meta-Analyses extension for Scoping Reviews (PRISMA-ScR) Checklist

## Data Availability

The dataset generated and/or analysed during the current study is available from the corresponding author on reasonable request.
