# Correction: Impact of the COVID-19 pandemic on breast cancer patient pathways and outcomes in the United Kingdom and the Republic of Ireland – a scoping review

**DOI:** 10.1038/s41416-024-02791-8

**Published:** 2024-07-25

**Authors:** Lynne Lohfeld, Meenakshi Sharma, Damien Bennett, Anna Gavin, Sinéad T. Hawkins, Gareth Irwin, Helen Mitchell, Siobhan O’Neill, Charlene M. McShane

**Affiliations:** 1 Queen’s University Belfast, Centre for Public Health, School of Medicine, Dentistry & Biomedical Sciences, Royal Victoria Hospital, 247 Grosvenor Road, Belfast, BT12 6BA Northern Ireland UK; 2 Northern Ireland Cancer Registry, Centre for Public Health, School of Medicine, Dentistry & Biomedical Sciences, Queen’s University Belfast, Mulhouse Building, Grosvenor Road, Belfast, BT12 6DP Northern Ireland UK; 3 Belfast Health and Social Care Trust, 51 Lisburn Road, Belfast, BT9 7AB Northern Ireland UK

**Keywords:** Breast cancer, Health services

Correction to: *British Journal of Cancer*; 10.1038/s41416-024-02703-w; Article published online 04 May 2024.

Following the original publication of the article, the authors wished to update the Acknowledgements section to highlight all contributions made to the article. The corrected Acknowledgements section can be found below, with the updated wording marked in bold. The remainder of the article remains unaffected.

ACKNOWLEDGEMENTS

Our thanks go to Breast Cancer Now, the research and support charity that provided funding for the “Impact of the COVID-19 Pandemic on the Diagnosis and Treatment of Breast Cancer” project, of which this scoping review is a part. We also thank **Miss Ann McBrien, a Metastatic Breast Cancer patient, PPI representative and member of the project Steering Group**; and Ms. Paula Darragh and Dr. Jamie Roebuck (Cancer Intelligence Officers, Northern Ireland Cancer Registry) for their work on the project.

The original article has been corrected.

