# Supplementary material for: Impact of the COVID-19 pandemic on breast cancer patient pathways and outcomes in the United Kingdom and the Republic of Ireland – a scoping review
Source: Br J Cancer. 2024 May 4;131(4):619–26. doi: 10.1038/s41416-024-02703-w (PMC11333579; doi:10.1038/s41416-024-02703-w)
Supplement: Supplementary file 1 — Table 1: Characteristics of 34 included studies [file 41416_2024_2703_MOESM1_ESM.docx]

Table 1: Characteristics of 34 included studies

| **Study ID** | **Published Date** | **Author** | **Research Design or Format** | **Study aim** | **Study location** | **Key findings** |
| --- | --- | --- | --- | --- | --- | --- |
| 1. | 2021 | [Armitage et al^9^](https://pubmed.ncbi.nlm.nih.gov/?size=100&term=Armitage+RC&cauthor_id=34461451) | Quantitative longitudinal study | To review the trends in adult national screening programme performance in England before and during the COVID-19 pandemic and consider the future implications. | England | A substantial reduction in performance was observed in four of the five national screening programs from 2019 to 2020. |
| 2. | 2022 | Borsky K et  al10 | Cohort study | To explore the hypothesis that the stage of breast cancer at initial diagnosis in 2020 is more advanced compared with 2019 | England | Average UICC stage increased from 1a in 2019 to 2a in 2020. |
| 3. | 2020 | [Cathcart et al^11^](https://pubmed.ncbi.nlm.nih.gov/?term=Cathcart%20P%5BAuthor%5D) | Communication | Impact of video-conferencing on the MDT was assessed by a survey to all participants. | England | Video-conferencing MDTs provide a solution to social distancing during the pandemic whilst maintaining a high level of patient care. |
| 4. | 2021 | Clark et al^12^ | Retrospective analysis | To assess the national impact of COVID-19 on the prescribing of systemic anticancer treatment in England, immediately after lockdown and after the introduction of new treatments to reduce patient risk. | England | In April, 2020, fewer registrations, in June, 2020, a 15% increase compared with the control period. |
| 5. | 2022 | Duffy et al^13^ | Observational study | To estimate the number of breast cancers whose detection may be delayed because of the suspension, and the potential impact on cancer deaths over 10 years | England | An estimated 148-687 additional breast cancer deaths may occur as a result of the pandemic-related disruptions |
| 6. | 2021 | Gathani et al^14^ | Observational study | Routinely collected NHS England Cancer  Waiting Time data were analysed to compare activity for breast cancer in the first 6 months of 2020 compared to the same time period in 2019 | England | A 28% decrease was observed in the number of referrals for suspected breast cancer, and a 16% decrease in the number of patients receiving their first treatment for a breast cancer diagnosis. |

| **Study ID** | **Published Date** | **Author** | **Research Design or Format** | **Study aim** | **Study location** | **Key findings** |
| --- | --- | --- | --- | --- | --- | --- |
| 7. | 2022 | Gathani^15^ | Communication | To compare routinely available national data for England, breast cancer service referral and treatment activity in 2020 and 2021 with those in 2019 | England | In comparison with corresponding periods in 2019, there were 16% fewer first treatments for breast cancer in the first half of 2020, 19% |
|  |  |  |  |  |  | fewer in the second half of 2020, but only 3% fewer in the first half of 2021. |
| 8. | 2023 | Gathani^16^ | Retrospective observational  study | To update breast cancer diagnostic services during the first 6 and 15 months of the pandemic after March 2020 | England | The overall number of referrals was 9% lower in 2020/21 and 9% higher in 2021/22 compared to 2019/20. |
| 9. | 2022 | Ho et al^17^ | Retrospective study of prospectively collected data from two cohorts | To compare the unit’s performance of microsurgical autologous breast reconstruction in the “post-COVID” period compared with the exact time period in the preceding year | England | In the post-COVID period, fewer deep inferior epigastric artery flaps were performed. |
| 10. | 2023 | Hudson et al^18^ | Natural experimental | To make use of a natural experiment to compare uptake in groups sent an open invitations with those sent a pre-booked timed appointments during a period when both invitation methods were in use. | England | During the period of the study (32.5%) women received a timed appointment and 67.5% received an open invitation. |
| 11. | 2020 | Joseph et al^19^ | Descriptive | To describe the rapid adaptations, we have made as a breast cancer service at a district general hospital in London in response to the pandemic. | England | Multiple changes in the breast surgery units were introduced e.g., staffing, stricter criteria for one stop clinic referrals etc, |
| 12. | 2020 | [MacInnes et](https://pubmed.ncbi.nlm.nih.gov/?size=100&term=MacInnes+EG&cauthor_id=32821623)  [al2](https://pubmed.ncbi.nlm.nih.gov/?size=100&term=MacInnes+EG&cauthor_id=32821623)0 | Prospective | To establish early surgical outcomes from breast cancer surgery performed during the peak of the COVID-19 pandemic. | England | A total of 202 patients underwent surgery in in the West Yorkshire region at the peak of the pandemic. Post-operatively, no COVID-19 symptoms were observed. |

| **Study ID** | **Published Date** | **Author** | **Research Design or Format** | **Study aim** | **Study location** | **Key findings** |
| --- | --- | --- | --- | --- | --- | --- |
| 13. | 2020 | Markeson et  al^21^ | Retrospective review of a breast cancer unit | To review the demonstrable early effects of service changes upon the unit and compare those to national and internationally published data | England | No immediate or delayed breast reconstructions between the start of lockdown (23 March 2020) and the end of May 2020. |
| 14. | 2021 | Purushotham et  al22 | Cross sectional | To examine the reduction in cancer diagnoses during the first wave of the pandemic and to examine the stage of diagnosis of patients with cancer presenting during the pandemic compared with that of patients presenting before the pandemic. | England | The number of new cancer diagnoses decreased by 18.2% from 2019 to 2020. In particular, prostate (51.4%), gynecological (29.7%), breast (29.5%), and lung (23.4%) cancer cases declined. |
| 15. | 2021 | [Shetty et al^2^](https://pubmed.ncbi.nlm.nih.gov/?size=100&term=Shetty+G&cauthor_id=34464568)^3^ | Retrospective observational study | To establish a triaging system for assessment of breast referrals from primary care to ensure safe and effective breast services without compromising breast cancer management. | England | The cancer detection rate remained similar at 4.2% of all referrals preCOVID and 4.3% during modified triage. |
| 16. | 2021 | Spencer et al^24^ | Population based study | To assess the impact of the pandemic on radiotherapy activity in England | England | In comparison with the same period in 2019, the mean weekly radiotherapy courses declined 19.9% in April, 6.2% in May, and 11.6% in June in 2020. |
| 17. | 2021 | Baxter et al^25^ | Report | To report real-time national systemic anticancer treatment delivery data from NHS Scotland | Scotland | The smallest decrease was seen in breast cancer (19.7%), which also had the most rapid recovery and the largest decrease seen in colorectal cancer (43.4%). |
| 18. | 2021 | [Campbell et](https://pubmed.ncbi.nlm.nih.gov/?size=100&term=Campbell+C&cauthor_id=34217418)  [al2](https://pubmed.ncbi.nlm.nih.gov/?size=100&term=Campbell+C&cauthor_id=34217418)6 | Descriptive | To describe the integrated approach to managing the impact of the pandemic on cancer screening programmes in Scotland throughout 2020 | Scotland | Early data indicates bowel, breast and cervical screening participation has increased since restart. |
| 19. | 2023 | Grocutt et al^27^ | Observational | to investigate the impact of the resultant contingency plans on radiotherapy cancer services in Scotland | Scotland | Total radiotherapy courses decreased (–10%) compared with the previous year, prior to the pandemic. |

| **Study ID** | **Published Date** | **Author** | **Research Design or Format** | **Study aim** | **Study location** | **Key findings** |
| --- | --- | --- | --- | --- | --- | --- |
| 20. | 2020 | Romics et al^28^ | Prospective cohort study | To evaluate the safety of breast cancer surgery during COVID-19 pandemic in a prospective observational study in the West of Scotland region during the first eight weeks of the United Kingdom national lockdown, and compared outcomes to the regional cancer registry data of pre-COVID-19 patients | Scotland | During hospital lockdown, tumor size was significantly larger in patients undergoing surgery. Approximately, 7.8% developed postoperative complications in lockdown. |
| 21. | 2022 | Bansal &  Saleem^29^ | Retrospective | To evaluate the pattern of primary care referrals to the symptomatic one-stop clinic during the pandemic | Wales | Ninety percent of referrals from the GP were urgent suspected cancers or  urgent referrals. This trend of referrals did not change over 2 years. There was a 5% and 7% cancer diagnosis rate in 2020 and 2019, respectively. |
| 22. | 2022 | Greene^30^ | Observational | To estimate the impact on incidence, stage and healthcare pathway to diagnosis for female | Wales | Cases decreased 15.2% (n = −1011) overall. Female breast annual IRR |
|  |  |  |  | breast, colorectal and non-small cell lung cancers at population level in Wales. |  | was 0.81 (95% CI: 0.76–0.86, p < 0.001), colorectal 0.80 (95% CI:  0.79–0.81, p < 0.001) and non-small cell lung 0.91 (95% CI: 0.90–0.92, p < 0.001). |
| 23. | 2020 | [Higgins et al^3^](https://pubmed.ncbi.nlm.nih.gov/?term=Higgins+E&cauthor_id=32487503)^1^ | Letter to editor | To summarise how COVID-19 has impacted local radiotherapy services | Wales | The overall attendance at the radiotherapy department declined by 28%. |
| 24. | 2023 | Mitchell et al^32^ | Observational | To quantify effect of policies on the diagnosis of three major cancers, comparing NZ with these two European countries | Northern Ireland | Comparing the pre-pandemic period to the pandemic period there were  statistically significant reductions in numbers of lung (23%) and colorectal (15%) PD cancers in NI and numbers of breast (18%) and colorectal cancer (18.5%) diagnosed in the NED. |

| **Study ID** | **Published Date** | **Author** | **Research Design or Format** | **Study aim** | **Study location** | **Key findings** |
| --- | --- | --- | --- | --- | --- | --- |
| 25. | 2021 | Bansal &  Chopra^33^ | Editorial | To evaluates the acute impact of COVID-19 on the provision of symptomatic breast services in the UK and explores suggestions for more sustainable functioning of services in the postCOVID-19 era. | UK | A more robust system of triaging  (via video link or telephone), Modification of one-stop clinics could be implemented. |
| 26. | 2021 | Dave et al^34^ | Cohort | To determine alterations to breast cancer (BC) management during the peak transmission period of the UK COVID-19 pandemic and the potential impact of these treatment decisions | UK | Among 3776 patients, 59% had their management altered by COVID. Endocrine therapy was used when theatre capacity was limited. |
| 27. | 2020 | Gatfield et al^35^ | Communication | To reassess patients receiving neoadjuvant or adjuvant systemic anti-cancer therapies for early breast cancer | UK | A majority of the patients were more reluctant to accept a therapeutic pause fearing cancer relapse than contracting COVID. |
| 28. | 2022 | O’Connor et  al^36^ | Quantitative study | To quantify the impact of COVID-19 on small biopsy procedures and cancer resection surgeries in the North-West of Ireland. | Ireland | An overall decrease in small biopsy (21.5%) and a decrease in cancer resection (14.4%). |
| 29. | 2021 | [Elghobashy et](https://pubmed.ncbi.nlm.nih.gov/?term=Elghobashy%20M%5BAuthor%5D)  [al3](https://pubmed.ncbi.nlm.nih.gov/?term=Elghobashy%20M%5BAuthor%5D)7 | Survey | To assess the effect of the COVID-19 pandemic on UK and Ireland-based breast pathologists to optimise working environments and ensure preparedness for potential future pandemics | Multiple sites  (UK &  Ireland) | Most breast pathologists (63.7%) have the option of working from home, and 36% have reported improved efficiency. |
| 30. | 2022 | Gannon et al^38^ | Observational | To investigate temporal and quantitative changes in radiotherapy regimens across England and Wales, using patient-level routine data to examine variation in use by patient/tumour characteristics as well as by geographical region, in women aged 50 years and over diagnosed with EBC | Multiple (England & Wales) | A striking increase in the use of 26Gy5F dose fractionation regimens for EBC, among women aged ≥50 years, within a month of guidance published at the start of the COVID19 pandemic in England and Wales |
| 31. | 2022 | Greene et al^39^ | Retrospective observational | To compare the 2020 and 2021 pathologically confirmed cancers with the 2019 pre-pandemic baseline in Scotland, Wales, and Northern Ireland. | Multiple  (Scotland,  Wales & Northern Ireland) | Between 2019 and 2020, the number of pathologically confirmed malignancies decreased by 14.1%. |
| **Study ID** | **Published Date** | **Author** | **Research Design or Format** | **Study aim** | **Study location** | **Key findings** |
| 32. | 2021 | Monroy-  Iglesias et al^40^ | Retrospective study | To report on the outcomes of cancer patients receiving radical surgery with curative intent during the first wave of the COVID-19 pandemic | Multiple  (England &  Italy) | Positive patients had surgery deferred until a negative swab. The radical surgeries declined by 6% as compared to the same period in  2019. |
| 33. | 2021 | [Perin et al^4^](https://pubmed.ncbi.nlm.nih.gov/?term=Puricelli+Perin+DM&cauthor_id=34217420)^1^ | Survey | To gather information about settings and assess decision-making processes that led to cancer screening suspension. | Multiple (England & Scotland) | The majority of settings (91%) suspended cancer screening services in March 2020 as a result of a decision by the government. |
| 34. | 2021 | Rocco et al^42^ | Virtual meeting | To evaluate how breast surgeons are adapting their surgical activity to limit viral spread and spare hospital resources | Multiple  (England &  Scotland) | A decline in the number of operating room schedules, the indications for surgery, and consultations were reported in addition to an increasingly restrictive approach to elective surgery. |
